# Trans narratives on school experiences—This is how we feel

**DOI:** 10.3389/fpsyg.2024.1373508

**Published:** 2024-04-12

**Authors:** Olatz Etxebarria-Perez-de-Nanclares, Maria Teresa Vizcarra Morales, Ana Luisa López-Vélez, Rakel Gamito Gómez

**Affiliations:** ^1^Psychodidactics Doctoral Program, Psychology of Education and Specific Didactic, Faculty of Education and Sport, University of the Basque Country, Vitoria-Gasteiz, Spain; ^2^Didactics of Musical, Plastic and Body Expression, Faculty of Education and Sport, University of the Basque Country, Vitoria-Gasteiz, Spain; ^3^Department of School Didactics and Organization, Faculty of Education and Sport, University of the Basque Country, Vitoria-Gasteiz, Spain

**Keywords:** trans people, school setting, segregation by gender, socioemotional wellbeing, inclusion

## Abstract

**Introduction:**

The school environment remains unsafe for many trans people, where they are victims of discrimination, aggression, and bullying, resulting in socioemotional and physical harm to trans individuals. Intersectionality and minority stress are contributing factors in this already challenging education environment. In many cases, the voices of trans people are not heard or listened to at school; therefore, this study aims to listen to their school experiences in order to identify key areas for improvement.

**Method:**

This study implemented a narrative research approach where six in-depth interviews were completed with trans participants from the Basque Country, Europe, and the United States of America that focused on five dimensions: being a trans, the role of school, lack of teacher education and training, segregation by gender, and socioemotional wellbeing. The transcriptions of these interviews were coded using Nvivo software in categorical systems in a deductive and inductive way.

**Results:**

The results clearly showed that the binary society has a negative impact on trans people. In addition, the educational environment is still hostile for most of them, in which the lack of teacher training and gender identity content in the curriculum has a negative impact on trans people and their experiences at school.

**Discussion:**

The findings support the idea that socially ingrained binarism is detrimental to all those who step outside the norm. It can also be said that the earlier the transition is made, the better the effect (clinical as well as socioemotional) on their personal lives. For this early transition to occur, it is necessary to have access to information from an early age.

## 1 Introduction

Discrimination based on sexual and/or gender identity is punishable by law in many countries, but the reality is that it is prevalent in our daily lives. The reason for this frequent and socially acceptable behavior might be that the majority of society is still cloistered in the gender binary (Di Marco et al., 2021). This cultural cisgenderism, as referred by Kennedy (2018), leads to the marginalization of trans and gender non-conforming individuals by making them invisible due to such tacit ideology. Moreover, they have been ignored in academic literature and the public consciousness (Horton, 2020). Kennedy (2018) also highlighted that cisgenderism, something that should be questioned (Martino and Omercajic, 2021), not only affects trans people, as it represents the male hegemony and misogyny that stems from it, but also affects everyone.

On the other hand, the difference between sex and gender is not very clear, otherwise it would not be possible to explain how sex can determine gender. This conflation is normative and exclusive, implying a binary system (Lindqvist et al., 2021), and this reality excludes the term gender diverse, which is understood to encompass those who identify with the feminine and masculine gender in combination, in variation, or neither of them (Tobin et al., 2022). It is important to note that, when gender is assigned culturally, the active agent is the other person which can lead to confusion and potential psychological or emotional harm to trans people (Kennedy, 2018).

Social identity, besides helping us to define ourselves, influences the social interactions we may have, and heteronormative socialization is believed to start from early developmental stages (Di Marco et al., 2021). Gender is a social construct, which, according to multiracial feminist theory, together with race, as well as providing identity, aims to provide principles of organization in the social system and maintain social hierarchy. According to social stereotypes, femininity has a bias of passivity and debility that is the norm for white women. The dominant heteropatriarchal cultures have created the image of the black woman with a clear contrasting effect. This image also reaffirms gender inequality among white people, making it seem that the weak white woman needs to be protected by the white man (Browne and Misra, 2003). Previous research suggests that transgender people have similar gender identifications before and after social transition for the reassurance of families who may think that the transition may take them away from an authentic identity (Call et al., 2021).

The condition of a trans person is a clear factor for having a poor social-emotional situation as stated in research (Etxebarria-Perez-de-Nanclares et al., 2023). They are exposed to victimization, bullying, absenteeism, low academic performance, family rejection, and harassment. Transgender and Gender Nonconforming (TGNC) students feel unsafe in the school environmentdue to verbal and physical assault. This situation can be understood as a result of institutionalized regimes of cisnormativity and cisgenderism (Martino and Cumming-Potvin, 2018), which causes harm and delegitimization due to trans-exclusionary curricula (Horton, 2022). Trans individuals' access to sex-segregated spaces and the ability to use the name of their choice are among their concerns (Bastian and Rohlik, 2022). Furthermore, trans men have the highest incidence of attempted suicide, which accounts for 51% (Tobin et al., 2022).

The socioecological model shows the importance of the impact of interactions between people and the environment on personal growth and development (Chan et al., 2022). Hence, the role that schools can play in people's wellbeing is relevant. The integration of families and teachers is important in this regard (Abreu et al., 2022) as a feeling of belonging is crucial. However, numerous studies have shown that one of the key barriers is the negative attitude and misinformation of educational professionals (Horton, 2020). On the other hand, educational protocols can help with the anxiety experienced by these students (Baum, 2022). In this regard, a need for engagement with trans studies is required (Martino and Cumming-Potvin, 2018). In addition, there are other elements, such as race, ethnicity, religion, and gender (even within the trans community), that can be the causes for multiple discrimination, meaning that socioemotional wellbeing can also be affected detrimentally by multiple aspects. Therefore, it is necessary to remember the factors of intersectionality as minority stress when assessing the wellbeing of this group.

Intersectionality is understood as the idea that multiple identity facets affect minority stress experiences (Call et al., 2021), mostly gender and race, it is possible to define minority stress as the social factors that create a negative social environment that can lead to high rates of psychological distress and poor mental health for those who belong to the oppressed minority groups (Johnson and Szilagyi, 2023). It is worth noting that black people have suffered decades of institutional racism with respect to housing, employment, healthcare, education, criminal justice, and financing (Tobin et al., 2022). In addressing intersectionality, it should be emphasized that those who belong to racially oppressed groups, cultural minorities, non-Christian, and lower-class groups experience higher levels of discrimination in educational institutions (Chan et al., 2022), which has a great effect on their socioemotional wellbeing.

Based on the factors identified in a systematic review of research articles with educational and social perspectives (Etxebarria-Perez-de-Nanclares et al., 2023), this study aims to listen to the different voices within the trans community in relation to their experience. Given the scarcity of studies that give a voice to the protagonists of this reality, there is a need for activist studies and student-centered interventions to improve the situation of specific students (Kirk and Oliver, 2014). Few studies even investigated from a perspective that is not clinical about trans people. Jerome Bruner highlighted how culture shapes the mind as it provides tools and context, understanding education as a process of negotiation between individuals and culture and highlighting the role of education in providing an alternative view of the world, making the strange familiar. He believed that engaging in narratives with others in socio-cultural interactions was vital in shaping the mind (Takaya, 2013). By giving trans people a voice, we learn from their experiences, identify difficulties, understand their perspectives, and can offer proposals in the educational environment to promote their freedom of development.

## 2 Method

### 2.1 Design

In this study, in-depth interviews were proposed to conduct ethnographic narrative research with testimonies as the fundamental pillar. To this end, the interviews were structured from the five dimensions suggested by Etxebarria-Perez-de-Nanclares et al. (2023) and were conducted in English and Basque.

### 2.2 Participants

In-depth interviews were conducted with six trans individuals, including four trans men (T3US, T4US, T5BC, and T6BC), a trans woman (T1BC), and a non-binary person (T2US), all aged between 20 and 29 years. Half of the participants (*n* = 3) were from the Basque Country (BC) (Europe) and the other half (*n* = 3) were from the United States of America (US), mostly from the state of Massachusetts. All interviewees were coded as T (trans), and each participant was assigned a number for identification purposes. The number one was assigned to the only trans woman and the number two to the only non-binary person as a sign of solidarity and criticism of this still sexist and clearly binary society. The rest of the numbers were assigned according to the date of each interview. The Basque participants transitioned during the middle or high school stages, while the US participants transitioned during the university stage. It is worth mentioning that T2US and T4US are currently teachers and that T1BC, T5BC, and T6BC are members of an association and deliver speeches in schools about their gender status.

### 2.3 Instruments

In-depth online interviews were the strategy used to collect data using the Zoom application. The interviews were conducted following previously set categories, which were renamed/modified with the aim of distancing them from a medical-clinical perspective and with the intention of giving a broader vision of trans reality, especially in the educational environment. The categories were determined as follows: being trans, the role of the school, the lack of teacher education and training, gender segregation, and socioemotional wellbeing. Field notes were also used to highlight the most significant findings and to collect additional information that appeared over the course of the interview.

### 2.4 Procedure

Due to the sensitivity of the subject and considering that these people were talking about their private lives, which in most cases have involved a lot of pain, the interviewer's personal and work circle was used to contact the interviewees through the snowball technique (Taylor and Bogdan, 1992). For this purpose, email, text messages, the WhatsApp application, and Instagram were used. The Basque Association of Families of Trans Children (NAIZEN) was also involved in contacting some of the participants. The interviews were recorded and were completed in Basque and English, and the captions tool (cc) was used for the interviews in English. The interview was conducted for a duration between 35 and 60 min. The objective of all the interviews was to build a strong rapport through trust and empathy, as interviews always aimed at understanding the other person, leading to a more detailed investigation (Denzin and Lincoln, 2015).

All interviews were transcribed using a word processor and uploaded to the Nvivo software program. Here, the categories described in Etxebarria-Perez-de-Nanclares et al. (2023) were created using deductive coding. Meanwhile, new subcategories were also generated from the information received during the interviews through an inductive procedure based on grounded theory. Table 1 shows the categorical system used, the subcategories, and the contributions of the interviews in each category and/or subcategory.

**Table 1:** A categorical system, the number of files, and the number of references.

**Dimension**	**Indicators**	**Files**	**References**	**%**
1. Being a trans		6	40	18.26
	What is it?	3	3	
	When did you realize it?	6	10	
	How did you realize it?	2	3	
	The concealment of the beginning	1	3	
	Transition and when	6	19	
	Doubts about certain decisions	1	1	
	Challenges	1	1	
2. The role of the school		6	72	32.88
	School climate	6	34	
	Gender identity in the curriculum	6	10	
	Decision making about the transition or in a school setting	6	21	
	Bullying	3	7	
3. The lack of teacher education and training		6	39	17.81
	When or where is this deficiency evident?	6	20	
	How can this problem be solved?	6	19	
4. Segregation by gender		6	33	15.07
	Segregated spaces	6	25	
	Safety issues	4	5	
	Sports	2	2	
	Uniforms	1	1	
5. Socioemotional wellbeing		5	35	15.98
	Discrimination	1	3	
	Negative experiences	5	28	
	Positive experiences	2	4	
Total			219	100

### 2.5 Analysis

Once the categorical system was created and all files were uploaded to Nvivo14, all paragraphs were read and classified according to this categorical system. The program counts the number of paragraphs (references) and from which interview they were extracted (resource). For the sake of accuracy, the analysis was supervised by three researchers.

## 3 Results

To analyze the results, five categories were defined, as presented in Table 1. For the purpose of the presentation of these results, excerpts in Basque were translated into English by the authors.

### 3.1 Being a trans

Trans identity is still not understood and/or respected in many social strata. The terminology has changed a lot in recent years, but there is no unanimity even within the collective. Although they were not asked about what it is to be trans, during the interviews, it was mentioned that binarism is very established in society, making it very difficult to be trans, and furthermore, in other cultures, such as in India or the United States with Native American, more than two genders are identified.

I think society is very binarized so ideas about trans people are generated about men or women and that's it. I think that makes it difficult to understand what it is to be trans. I think that binarism limits and that when we get out of it, everything will change (T1BC).

It is important to highlight the criticism made toward society, making it clear that trans women, for example, are not another type of woman, and emphasizing the necessity to move away from a clinical approach.

…trans women are not different type of woman (T2US).

Being transgender is identifying as a different gender, but you can go through those surgeries in those medical transit. It goes under the idea that to be trans you don't need those, you are who you are and what you decide to do with your body is your own business (T3US).

This finding makes it clear that gender is a social construct, not only because in different cultures the classification of gender is different but also because the meaning of being a woman, or a man, is not the same in all cultures. Moreover, it is striking that, although a part of society is reluctant to move away from binarism, it is the same society that classifies trans women or trans men into other types of women or men.

All the interviewees discovered their trans status in middle or high school, and only one mentioned that he knew he was a boy since he was 6 years old. We can observe a lack of information about gender identity that hindered this “egg cracking” moment in this journey toward discovering their own identity and how the lack of safe spaces or adequate modeling delayed taking the step.

Oh, man, that was a journey of itself. I started exploring my identity when I was around 14 just getting into high school. It was a bit of me trying some things out at first, it was kind of a pipeline, of one identity to the other, until I eventually fell into the identity of well, more masculine presenting […]and the day that I kind of what they call it is like an egg cracking moment, where it's like dawning realization like, it's like step by step, and then, there's is like a “Oh, that's when that realization came”. But for me, when I finally realized that it was the more masculine leaning that I was more comfortable with, I just gotten out of the shower and I was looking at myself in the mirror, and it was just a moment where I said he, and I was like, “Oh, there it is” (T3US).

It is evident that a lack of knowledge makes trans people discover their own identity later. Even discovering it at an early age, the lack of information, safe spaces, and adequate referents can make these individuals think that what they are feeling is not right.

In this sense, it is also mentioned that the thing that led them to realize what was happening was the discomfort they felt.

You know that something is wrong (T6BC).

This type of situation can lead to a denial of what is happening and have a huge negative impact on their self-esteem and consequently on their own development as a person.

In the end, I spent 9 years, well, probably more because I realized I was a boy when I was 6 but before I noticed that something was not right, that there was something that differentiated me from others. And that had a big impact on my self-esteem and self-concept, because if you spend years thinking that you are crazy, that no one will love you as you are… Also, it's a secret, you can't talk to anybody about it, because you think nobody will understand you, and that had a big impact on my head (T5BC).

Being different is something that society punishes. Therefore, is it necessary to transition? What emerged from the interviews is that it is a personal and non-transferable process as each individual is unique. Not everybody understands the same thing about transitioning and has the same needs and, often, the fact of having to transition puts extra pressure on trans people. It also invites a reflection on what transitioning really is and whether it is the trans person or society who in fact transitions.

I believe that people still think that we are the ones who make the transition, but no, I have always been a boy, what has changed has been the way society perceives me, we have always been what we are […] But my goal is to make the transition a natural process (T5BC).

That is, hard to say because it is also personal to an individual, I mean what I think of when I hear that question is a little bit unfair, just like the way Society is set up. The question must be posted like that, but it's like, why does it have to be that I have to do this whole sort of like performative thing where I'm like revealing to you who I am, and that it's different than what you thought your whole life? That whole thing is a bit of a dramatic thing. I mean, the rate ideally and ideal world it would be that everyone understood you know, everyone has pronouns […] Or it is like “oh, man!” I must be consistently worrying about coming out and I'm going to come out and if I should. And how people are going to react? Because it is this whole, like, big societal thing (T2US).

It is necessary to transit if you think it is appropriate. Transition for each person is something different. For me, the transition is to understand the ideas in your head or to develop and understand the identity […] it is like the skin, each one has its own, with which we go out into the world. I believe that transition is a tailor-made suit (T1BC).

Similar answers were obtained when asked when the transition should take place. There was fear of not only hormonal treatments at early ages but also the prevalence of a lot of misinformation. It was also mentioned that it seems that doing hormonal treatment at an early age has many advantages.

In my mind, I do not think that I was ready as a teenager… But at the same time, I have heard of like very young children being given hormone blockers, so that they don't have to go through the first puberty to even begin with, which sounds like deal to me because once you go through your puberty, and you start transitioning, after that you have to go through puberty again. So if you can stop it for a second and be able to think about what kind of puberty do you want to go through, then maybe you only have to go through one […] I did not start taking hormones until after I graduated, because I was afraid that the hormones would affect my ability to graduate, bullshit (T4US).

… knowing yourself for the sooner, the better. Yes, absolutely, that is why talk about this, education. As like a core, standard, and all that, like I am talking like from pre-k (T2US).

Any time is a good time. When you know who you are and want to show it to the world. It is true that in the school environment, when you are younger, it is much more amiable. The older you get, the people around you have more fixed ideas about the world around them, which makes it more difficult (T1BC).

Although it is unclear whether they are in favor of transitioning in public or not because it is mentioned that it can also be harmful, when they talk about their personal experience, they value it positively, although, in one of the cases, it is expressed that transitioning was the only alternative to suicide.

I myself this is one of the best things that ever happened to me, transitioning. I was a very, very angry child. Now I have anger, but it is nothing compared to what it was. I am much happier than I have ever been. I have pride in my beard in my appearance. Yeah […] I was a lot angrier before I transitioned, and then transitioning actually mellowed me out. It is going to sound so dramatic, I didn't know happiness before but now I am pretty happy, happy as I have ever been in my life (T4US).

I made the transition because it was the only alternative to death since I wanted to die. At that moment something changed in my head and I thought the consequences of transitioning cannot be as bad as death. Therefore, my goal is that people do not reach that point of wanting to die (T5BC).

There is a reflection regarding society about transition in which it is not clear which part of the transition is performed to comply with one's own desires or to comply with what society demands of them. As well as how the transition can modify your social environment, for example, from being surrounded by women when he was perceived as a woman to them not feeling so comfortable with him since he started being perceived as a man.

### 3.2 The role of the school

The school is where we learn social rules, interact with peers, and feel belonging to a group, which is the key to the developmental stages. Due to this social aspect, the negative experiences suffered in the school environment have a great impact on the perception of their self-image, self-esteem, and, consequently, on their emotional wellbeing and full development.

Most of the interviewees reported negative experiences in the school environment.

I did not feel a nice and safe environment in High School. It was a very big high school and I am sure there was plenty of people who accepted it, but there were some like I definitely got some harassment […] I still hear kids say like you are gay as an insult. I have also heard kids say that and then seeing a teacher do nothing about it and that is a problem, too (T4US).

In general, I have never experienced school as a safe environment. I had a lot of problems going to school, I got sick because I didn't want to go, it was hell. When you feel that you are different or you are left out…. I also think that especially in high school, not enough importance is given to emotional wellbeing. The teachers focus on academics, but a student can't focus on studying when they want to die. That is why I think it is also the teacher's responsibility to accompany them, to ask how they are doing (T5BC).

Even if you don't want it to, society influences you, what is expected of you. Sometimes I would like to be aware of which steps I took because I wanted to and which because of what was expected of me. There are people who think that it is a whim of ours, and many times it is the answer to what is demanded from outside because I believe that for society there is a when and how to transition (T1BC).

Not all experiences were negative, there have also been those who have experienced a friendly academic environment, in which safe spaces have been key.

Before I began the transition, I changed high schools, and the environment was totally different […] I didn't suffer any aggression, but if someone said something inappropriate, the teachers were very attentive and cut it right away […] They also started giving talks on the subject at all grade levels. Then more trans people came out and today it is a reference school for trans people […] The truth is that my opinion about the school has changed after my transition, I had a very nice transition (T5BC).

The school that I went to was very accepting of different identities. It was at art school, it is a bit more diverse […] Everybody around me was being very open about it. My school did have a LGBT club. It was kind of like another art club but it made a safe space for a lot of kids that I think it is very important, because they might not have that at home. The new art teacher made a club for us, just a nice little place to go and hang out […] Those kinds of spaces are very important to have, I think (T3US).

It can be inferred from the interviews that teachers play a very important role in the perception of school climate and that they are the agents who have the power to balance the scales for better or worse. In addition, it is important not to lose track of one of the main objectives of the school, which is to protect all students.

The best way to protect children is to educate the people around them (T5BC).

When asked about gender identity within the curriculum, most of them did not receive any information within the educational environment, and even those who did receive it believed it was insufficient. It was highlighted that, if they had that information, they would have discovered their own identity earlier.

My school and High School, yes. We have always had training on this topic for both teachers and students. In tutoring hours, we could choose the topic we wanted to talk about, and this topic came up more than once (T6BC).

What I have learned has either been through my own research or through my friends. I have never learned the lick of anything about LGBTQ identities, and what they mean, and how to approach them through any kind of school curriculum […] I think it would be really important to have those curriculums on like gender identity, especially in like health classes. If I had a health class that talked about like different identities through gender, “Oh, my God!” I probably would have had that realization way sooner (T3US).

As to whether they consider school a suitable place to transition, there is no agreement. The need for knowledge and understanding on the part of the educational community is also mentioned.

But I mean, no, school is hard for everybody. I think and anything that kind of makes you different makes it a little bit harder. And right now, you know, trans is the new gay (T4US).

I think it would help a lot of kids or a lot of teenagers who are trying to figure themselves out that they know that there is a place to turn to learn more about it because that is what school is about (T2US).

The school setting is not a requirement to have those kinds of realizations, but it is still a good place to start, because you, as a child, you learn more about yourself through your peers (T3US).

When the need for protocols is raised, although they are in favor of them, they highlight more important factors such as listening to and respecting trans people and training on the part of the educational community. In some cases, the school counselor is mentioned as being responsible for this process.

Protocols, of course, help, but it is more important to listen to our wishes and needs […] Our needs and opinions should be above the protocols […] I think they should be broad, covering all areas, but being aware that it is something that should protect us and that it is a foundation (T6BC).

I think that that should be the school counselor, but their knowledge should not just be based of textbooks, either (T4US).

Bullying continues to be one of the worst scourges to affect the education system. Although there are protocols and laws that regulate the prevention and procedures for these cases, it still happens daily and in the case of trans people, there is a higher probability of it occurring.

I definitely was bullied about my sexuality in school, and if you see pictures of me from like fourth or fifth grade, I looked like a little boy, and people used to think that was an insult that they called me man. “You look like a boy.” Okay… So, thank you. […] I guess I have experienced both gender and sexuality (T4US).

### 3.3 Lack of teacher education and training

All the interviewees agreed that education professionals do not have sufficient gender identity training to respond to the needs of trans students, in particular, and all students, in general. When asked about when or where this lack is evident, they coincide in saying that they do not know how to proceed when a problem arises, giving a delayed response, and even, on occasions, no response at all.

It is also mentioned how the professionals, the counselors in this case, were not aware of the current legislation regarding gender identity.

I did have problems with people using slurs about my sexuality and someone straight out told me right in front of a teacher I knew over he is also gay “No, I just I do not like gay people, I think they're disgusting,” and I am looking at this teacher like “You're really not going to say anything about this? Nothing to said […] I am also a teacher who has had transgender students as well […] One of our counselors wanted to out a student to his mother, so I went to the principal and she pulled up the State of Massachusetts' policies and sent them to that counselor. Policy states that well, we have to call them their names, and the pronouns that they want, and we cannot tell their parents until they give the permission […] I just finished a master's in the teaching field, and that was never addressed […] If teachers know something about it is because they have taken the initiative to go out and educate themselves (T4US).

Adults are hearing a lot of stuff throughout the day, and they are not responding in the way that they should be (T2US).

Later, it became clear that they had no idea how to manage, for example, the issue with changing rooms. They told me to use the spaces I wanted but they were not aware of the weight that society has on gender identity […] My identity is built around others. (T1BC).

When asked about how this lack of training could be addressed, many voices claimed the need for real testimonies of trans people, justifying that their testimonies would make the training more relevant. There is also a need for training on diversity, where sexual orientation and gender identity should be included. In addition, it was expressed that the issue of training should not rely on teachers and governmental institutions, instead, it was implied that the responsibility lies with school districts to address this drawback.

I think there should be a diversity class, and the teachers' training programs, and that diversity class should include gender identity, gender expression, sexuality, as well as race, and ethnicity. I think like often diversity in schools means race and ethnicity, and no so much sexual orientation and gender. Sort of like “It is not ok to talk it in school,” which is ridiculous (T4US).

### 3.4 Gender segregation

As a reflection of a clearly binary society, there are spaces of daily use, even related to basic needs such as restrooms, that are segregated by sex.

Among the interviewees, there are those who have not experienced using the restrooms as a traumatic experience. As for the solution to gender segregation, there is a disparity of opinions. Some think that restrooms should not be segregated by sex, that they should be descriptive only and state what they contain, and others propose a third option that rather than being an exclusive restroom for trans people, it should be gender neutral or otherwise, it would segregate them. It is also mentioned that enabling the use of the school nurse's restroom may suggest there is something wrong with the person. At the same time, enabling the teacher's restroom for this purpose has been positively evaluated.

I would always flip-flop between using the woman's and the men's restroom […] I do think a gender-neutral bathrooms are a really good idea […] It is just a bathroom, I just want to go pee that is it […] As like Gym Locke rooms goes, I think that is a bit of a trickier one. […] There should be those private stalls so people still are not having a crisis every time they need to go change (T3US).

I didn't feel comfortable changing with boys. With girls it wasn't so much because of my identity, but because of dysphoria […] Being trans, you go from being normal to being weird […] I would go into the locker room earlier or later […] I think we should normalize the mixed option, but there has to be an option where we all feel comfortable and safe […] For me, a third option can also be a good thing, but not a third option just for trans people, to segregate, but to create a new safe space (T1BC).

The COVID baths case (restrooms designated for people who could be infected during COVID-19) shows that if there is a will and/or a need, it is possible to find other formulas.

While some argue that the lack of gender segregation in restrooms can generate safety problems in both directions (cis women and trans people), others defend that the restroom is only a place to relieve oneself without any other consideration.

I think what people do not realize is how much violence like trans people are subjected to […] When we talk about bathrooms, about that safety thing because of how that violence, [sic] violent language that people like put around. It is their perceptions. Really it shows, it reveals society's perception of men that they are freaking bite [sic] and aggressive and that's the way that we're socializing them. So, that is a whole other problem that needs to be dealt with (T2US).

As far as the bathrooms go, that was an idea struck up by people, we call them TERFs, acronym for Trans Exclusionary Radical Feminist, and it basically puts forth the idea that, well, it is feminism under the guise of blatant transphobia, where men are trying to get into women's spaces and under the guise to hurt them. Where trans women are just being discriminated because they are trying to invade those spaces when that is not the case (T3US).

The issue of school sports is also mentioned when talking about segregated spaces since most of them are separated by gender. One of the interviewees quit soccer because he played on a girls' team, opting instead for boxing. Not only have group sports been a problem, with activities such as swimming, where a greater proportion of the human body is visible, also generating discomfort.

I was probably the first time I experienced people having maybe like a high confidence, or like low shame for their body. I do not know if they are feeling just like very sure of themselves, or whatever but I was definitely not one of those people who was getting naked at gym, and Locke room […] Yeah, never did swimming, or anything like that (T2US).

School uniforms can also be controversial, not only because they clearly reflect the prevailing binarism in society but also because they are evidence of sexism by a certain section of the population.

Even something like getting dressed, I mean in Springfield there is a Uniform Policy. So, the whole district is in uniforms. And I remember like challenging an administrator, who is telling me to go home because I have on a skirt, that he had been too short, and there was a traditionally male looking whatever student who had walked in directly before me who had shorts on who that were so short [sic]. It was just one of those gender things. Why isn't it appropriate for me, but it is for him. Like what are we saying about gender? (T2US).

### 3.5 Socioemotional wellbeing

Their wellbeing is negatively impacted by the mere fact of belonging to the trans community. However, it should be noted that certain groups related to race, ethnicity, religion, etc., are discriminated against furthermore, which has a greater impact on their emotional wellbeing. Therefore, intersectionality and minority stress are factors to be considered.

I have not talked about it on this call, but like the intersections of race, with all these things, just exacerbate everything to the tenth degree. Like how many, for example, black trans people are killed every day. That is the violence we should be worried about. And I feel like it is not like an individual thing that has to do with the media […] It is also incredibly depressing to have trans people and specifically black trans people, dying at such an exacerbating rate. This creates a storm, I do not know, like discomfort […] Why when people talk about privilege, some people just have the privilege of not having to be hyper aware about what is happening around them because they are cis, or they are white people or they are Christian, or they are whatever? It is on that binary skill that means that they are safe from the violence of white men (T2US).

It can be extracted that not being aware of race, sexual orientation, or gender identity is a privilege of white cisheteronormative society. The negative experiences of this group are various and occur on a daily basis, where they experience insults, aggression, contempt, school absenteeism, and even suicidal thoughts.

I was holding my girlfriend's hand walking down the sidewalk and high school, and someone behind us decided to call us fag […] I have actually heard some of the students that we work with at the school use that word, and every time I hear it, I give a history lesson. What that word means is a bundle of sticks, and what you're saying when you say that to a gay person is that you believe because they're gay they should be let on fire and die […] What doesn't kill you just makes you stronger. So that's kind of how I look at (T4US).

My mental health has suffered because I was not given proper support at that time. I didn't grow up in a very accepting household […] A lot of that affected my communication skills, I would have trouble communicating what I wanted […] School was essentially my escape. A lot of the times I didn't want to go home because I was just getting very bad there […] I was not respected in my household, and the only place that I was, was in school with my peers. But it is certainly as far as mental detriments not being able to be given a proper outlet to communicate those kinds of things, it very much detriments in a developmental way (T3US).

For our mental health not to be affected, it is necessary that our identity is accepted, that our identity is visible in society, and the first step should be that we are respected as we are. Because otherwise you feel annulled as a person (T1BC).

However, it can also be said that there seems to be a small change for the better in society, as some have had positive experiences throughout this journey. The geopolitical factor is also important, as there are areas that are more accepting of gender diversity.

I am from a very small town, and I don't know if that has played in my favor or if all the stars have aligned, but I have never had any problems […] I think I had the problem when I didn't know what was wrong with me. When I knew I was a boy, all my problems disappeared. That's why I say that being trans is not the problem, but the solution […] I think that, in the Basque Country, the reality is much kinder to trans people (T6BC).

## 4 Discussion

### 4.1 Being trans

According to the testimony of the six people interviewed, deep-rooted social binarism may be a reason why trans identities are not accepted in society. Gender roles and norms are a social construct (McBride, 2021), and performing outside of what is socially established is punished, which may be a consequence of cultural cisgenderism (Phipps and Blackall, 2021). In fact, feeling different from what is socially established is often the beginning of the journey toward finding one's own identity as some of the participants mentioned. In the interviews, the importance of knowing adequate referents is mentioned to facilitate the search for identity, which is described as a continuum until disclosure. The association that each individual makes with the term transitioning is also different. For some, transitioning means taking the medical/clinical route, whereas for others, it is socializing their gender identity, and for others, it is society that transitions. Many prefer not to make their gender identity public because of the consequences and feel safer following the norms of cisheteronormative society. Thus, trans identity, like any other, is as unique and non-transferable as the skin that covers the body of each person (Di Marco et al., 2021).

Among those interviewed, there is unanimity that transition should be made when the person is ready, but everything points to the fact that the sooner it is done, the better the results at all levels, since children learn more easily, naturally, and with greater tolerance. Unlearning is a much more complicated process than learning itself, and there is evidence that children who socially transitioned in early stages (prepubertal) have similar levels of depression and moderately higher anxiety levels compared to their cis peers (Olson et al., 2016).

### 4.2 The role of the school

The majority of the trans people interviewed have experienced a bad school climate where they were teased and verbally attacked because being different is punished. Martino and Cumming-Potvin (2018) express that there is a need to address gender justice in education. Schools are not only responsible for instilling academic knowledge but also for working on all the transversal knowledge that helps us to live in society and to be respectful and tolerant. Research shows that hostile school environments worsen educational outcomes (Call et al., 2021; Feijo et al., 2022; Johnson and Szilagyi, 2023), and academic professionals play an important role. In addition, the creation of safe spaces, such as Gender Sexuality Associations or Gay-Straight Alliances (GSAs), may be key in the perception that trans people may have about the school climate, improving connectedness and decreasing the risk of suicide (Baum, 2022; Chan et al., 2022; Feijo et al., 2022; Marraccini et al., 2022; Tobin et al., 2022; Johnson and Szilagyi, 2023). Besides, the training of the professionals in charge and the educational community in general is of utmost importance, since, without specific knowledge, educators will be unable to protect their students or provide an adequate response to their needs. One of the main objectives should be to create more positive and inclusive learning environments for these minority groups, emphasizing that a school needs to be protective, stable, inclusive, and pleasant for all students (Chan et al., 2022). The same study points to the need for a critical pedagogy to teach young people about the negative impact of any kind of discrimination.

Gender identity is, as the participants stated, not present in the educational curriculum in most cases. The incorporation of gender identity content beyond the binary system could serve as a crucial factor not only in improving the school climate but also in preventing many unpleasant situations among peers and fostering interaction with the rest of the educational community. This knowledge would help many other people to identify what is happening to them and thus provide appropriate modeling, giving rise to a trans pedagogy of refusal (Martino and Omercajic, 2021). It is clear that visibility and representation are needed (Horton, 2020) In fact, 27 qualitative studies confirm that exposing children in elementary school to gender diversity makes them challenge gender norms and become more flexible (Johnson and Szilagyi, 2023). On the contrary, studies show that trans people discover vocabulary related to this world at the age of 15 years and 6 months, meaning that they are unable to express themselves until this time, with all the possible consequences this may have (Kennedy, 2018).

The need for protocols for an adequate transition is seen as necessary by the participants and the existing literature (Kurt, 2017; Meyer and Keenan, 2018). However, it is stressed that it is much more important to listen to people, since everyone has their own path, although gestures of humanity, such as understanding and respect, can help more than any protocol, in line with that extracted from the interviews. According to the interviewees, inclusive language can be another small gesture from the educational community, and this affirmative communication is being prioritized in most recent school guidance documents (Horton, 2020). This form of communication may have an impact on eliminating the bullying that is present in all educational environments, as this group is one of the most persecuted. It is important to look after the welfare of trans people during activities sponsored by the educational community even if they are outside school premises, as they also influence school climate (Baum, 2022). It is therefore necessary for schools to recognize their transphobic practices, retrain their staff, and reformulate a curriculum where gender diversity is addressed (Feijo et al., 2022; Marraccini et al., 2022; Johnson and Szilagyi, 2023).

### 4.3 Lack of teacher education training

The respondents believe that education professionals do not have enough training on gender identity to support these students and give an adequate response when uncomfortable and even unacceptable situations arise. This lack of training is especially noticeable when a problem appears. It is necessary to train these professionals and research suggests that this could be done when educators are attaining their degrees (Davy and Cordoba, 2020; Bastian and Rohlik, 2022). Teacher attitudes, knowledge, and confidence play an important role in inclusion (Horton, 2022), but some professionals are afraid to discuss certain issues in the classroom because of the reprisals they may suffer from superiors and/or families. This fear is also reported by Abreu et al. (2022).

Based on their experiences, some of the interviewees suggest that, in recent years, the situation has changed, but there is still a long way to go. There is a need to reflect on what diversity is, alluding to the fact that, to date, it has focused on race and ethnicity. It is necessary to consider including under these umbrella elements, such as gender identity, sexual orientation, and physical and cognitive disabilities, as it is also mentioned that there are higher rates of gender diversity among children with neurodevelopmental conditions such as autism spectrum disorder (ASD) and attention-deficit and hyperactivity (ADHD) (Call et al., 2021).

### 4.4 Gender segregation

Gender-segregated spaces continue to be another challenge that must be addressed not only in the educational environment but also by society in general, as trans people tend to avoid these spaces due to fear of harassment (Feijo et al., 2022), something also enhanced by the TERF movement (Harris et al., 2022). The choice of these individuals regarding which spaces they want to use must be respected, as it has been shown that gender-affirming policies reduce these barriers to wellness and encourage Transgender and Gender Diverse (TGD) youths to participate more in sports activities when they are allowed to use the locker rooms of their choice (Call et al., 2021). In contrast, lack of access to gender-affirming restrooms and locker rooms leads to a greater probability of suffering sexual assault (Johnson and Szilagyi, 2023).

The segregation of restrooms and uniforms can be understood as another form of cisheteronormative violence (McBride and Neary, 2021). The binary formula is insufficient and not very inclusive, and creating a third option only and exclusively for trans people would further accentuate gender segregation. The subject of sports also generates controversy, since there are no clear guidelines on how to proceed. Research carried out in the sports medical field concludes that the physical superiority of men over women depends largely on being or having been exposed to the male hormone (testosterone), and it may represent a 15%−30% difference between men and women, suggesting that, at least until middle school, there should be no problem for these students to participate in any of the gender-segregated groups or all together. There must be a response from society to this group regarding this issue, especially considering that participation in sports activities is closely related to higher self-esteem, lower levels of depression, and a sense of belonging in school (Johnson and Szilagyi, 2023).

### 4.5 Socioemotional wellbeing

The impact that the gender identity condition has on the socioemotional wellbeing of trans people is evident in the interviews conducted in this study, as this collective is considered to be one of the most oppressed and marginalized (Davy and Cordoba, 2020). It should be considered that certain hormonal treatments may involve pathophysiological alterations of the depression spectrum and eating disorders (Manonelles et al., 2023), although these alterations would not justify many of the situations experienced by the interviewees.

The theoretical framework of intersectionality and some of the responses from the interviewees highlight the role structures such as racism, sexism, heterosexism, and classism play as power relations in experiences at the individual level, particularly for those who are marginalized at multiple intersections, and how research, healthcare, and education can shed light on ways to mitigate mental health disparities among TGD youth (Bowleg et al., 2023).

All of the above conclusions derived from interview narratives show that schools must respond to trans students by making the educational environment safe and friendly for all students. The creation of safe spaces can be of great help to trans students. There is an urgent need for training in gender identity for all education professionals, and gender diversity should be made part of the curriculum, as training and information will be key to diversity and the real inclusion of this group. Inclusive language and zero tolerance for verbal aggression will help in this direction. Sex-segregated spaces continue to be a problem for trans people, thus a reformulation of these spaces in the educational environment is necessary. A third option that is not exclusively for trans people could serve as an option. All these implications will help the socioemotional wellbeing of these people to improve considerably.

## 5 Limitations

This study consisted of six participants, five of which were white, and there was only one representation of a trans woman and one of a non-binary person. The limited research on non-binary people is evidence of a highly binary society, and more specific research on non-binary individuals is still needed. In addition, the study focuses on middle-class white people, with a lower representation of people from minority groups and/or people with disabilities.

The lack of trans authors or authors of color has also restricted our scope of inquiry, further reinforcing the existing dominant white origins of the academia in our study.

## Data availability statement

The original contributions presented in the study are included in the article/supplementary material, further inquiries can be directed to the corresponding author.

## Ethics statement

The studies involving humans were approved by the Ethics Committee of the UPV/EHU and favorably assessed, obtaining the ethical treatment code TI0263. The studies were conducted in accordance with the local legislation and institutional requirements. Written informed consent for participation was not required from the participants or the participants' legal guardians/next of kin because there are interviews recorded where they express their consent.

## Author contributions

OE-P-d-N: Writing – original draft, Writing – review & editing. MV: Writing – review & editing. AL-V: Writing – review & editing. RG: Writing – review & editing.
